# Income Inequality, Income, and Internet Searches for Status Goods: A Cross-National Study of the Association Between Inequality and Well-Being

**DOI:** 10.1007/s11205-015-1158-4

**Published:** 2015-11-02

**Authors:** Lukasz Walasek, Gordon D. A. Brown

**Affiliations:** Department of Psychology, University of Warwick, University Road, Coventry, CV4 7AL UK

**Keywords:** Income inequality, Conspicuous consumption, Status seeking, Consumerism, Google Correlate, Google Trends

## Abstract

Is there a positive association between a nation’s income inequality and concerns with status competition within that nation? Here we use Google Correlate and Google Trends to examine frequency of internet search terms and find that people in countries in which income inequality is high search relatively more frequently for positional brand names such as Prada, Louis Vuitton, or Chanel. This tendency is stronger among well-developed countries. We find no evidence that income alone is associated with searches for positional goods. We also present evidence that the concern with positional goods does not reflect non-linear effects of income on consumer spending, either across nations or (extending previous findings that people who live in unequal US States search more for positional goods) within the USA. It is concluded that income inequality is associated with greater concerns with positional goods, and that this concern is reflected in internet searching behaviour.

## Introduction

Income inequality is associated with a number of severe social, psychological, and economic indices of reduced well-being in societies (Kondo et al. 2009; Wilkinson and Pickett 2009). Although this relationship appears to be robust and has been demonstrated at both national (e.g., US, UK, Germany) and cross-national levels, its exact causes are not well understood. One possible psycho-social mechanism underlying the link between income inequality and societal ill-being is based on the social rank hypothesis, which maintains that income dispersion determines how much attention people dedicate to their income-related social status (Brown et al. 2014; Daly et al. 2015; Walasek and Brown 2015). When large income gaps separate the poorest and the wealthiest in a society, income becomes a more accurate indicator of one’s status (social rank). Consequently, in order to increase their rank position in the income distribution, people rationally devote more effort towards status competition when they live in more unequal societies. The urge to “keep up with the Joneses” is expressed partly in higher interest in positional goods (Hirsch 1977), which function as a signal of higher income and wealth. The social rank hypothesis maintains that societal well-being suffers when people put social status ahead of other important aspects of their lives, such as their family, traditions, or maintenance of other supportive and health-protective relationships. In turn, status competition (or status anxiety; Layte and Whelan 2014) is identified as an important cause of poor health and well-being in a society.

While many classic economic models of consumer demand fail to acknowledge the role of status competition (Chao and Schor 1996), recent evidence supports the notion that consumption patterns are different in unequal societies. When income inequality is high, people save less and spend larger portions of their disposable income (Alvarez-Cuadrado and Attar 2012; Cynamon and Fazzari 2015; Heffetz 2011). For example, using data from the German socio-economic panel, Drechsel-Grau and Schmid (2014) found that the poorest spend more when the earnings of the wealthy people increase. In order to spend more, people tend to work longer hours (Bowles and Park 2005), but yet are more likely to become indebted and go bankrupt (Perugini et al. 2015). What is it then that people spend their money on?

Evidence from economics suggests that inequality leads to increased consumption of status (or positional) goods. Bricker et al. (2014) found that the rank position of a household’s income among its neighbours was a strong predictor of the quality and value of the car that the household owns. Bricker and colleagues propose that, in an attempt to compare more favourably against others, people are willing to spend more of their income on newer and more luxury vehicles. In a similar vein, Chao and Schor (1996) found that large gaps in income distribution determine preferences for luxury brands of cosmetics. In the presence of high income inequality, people purchase more expensive brands of perfumes even when the correlation between their quality and price is low. Heffetz (2011) analysed income-demand elasticity of various goods as a function of their visibility. Using a large telephone survey, Heffetz identified a list of durable and non-durable goods that are most easily noticed when owned by others. Consistent with the social-signalling account, goods that are most visible, and therefore signal social status better, were shown to have the highest income-demand elasticity.

Purchasing is one behavioural index of individuals’ concerns with positional goods, but is limited in a number of ways. First, expenditure can be seen as an outcome measure which reflects underlying concerns (as hypothesised by the social rank approach), rather than being itself an indicator of the amount of time and mental resources being devoted to status competition. Secondly, and relatedly, mere purchase of positional goods does not in itself indicate the level of cognitive effort that individuals are devoting to researching and considering their purchases. Thirdly, purchasing data carry no information about the concerns and values of individuals who may not be able to afford the positional goods they would like to possess.

To address these concerns, Walasek and Brown (2015) examined internet searching behaviour as a function of income inequality in different US states. Specifically, they used relative search term frequency to gain an insight into societal concerns and values. The authors used Google Correlate (https://www.google.com/trends/correlate) to obtain a list of internet search terms whose relative search frequencies correlate most positively (and negatively) with state-level income inequality. In order to examine the effect of income inequality after controlling for other variables, the authors first obtained residuals from regressing income inequality (GINI coefficient) on various control variables, including log of mean income, state population, percent of foreign born population and percentage of urban population. These residuals were then used as input for Google Correlate. The results were consistent with the social rank hypothesis, in that the search terms found to be used more frequently in states with high income inequality were largely concerned with status goods, such as designer brands or expensive jewellery. At the same time, none of the search terms that were most negatively correlated with inequality were related to positional goods.

Here we extend the internet search methodology used by Walasek and Brown (2015) in a number of ways to better understand the relationship between income inequality and people’s concern with positional goods. First, using country-level data on income inequality and internet search frequencies, we examine whether the results reported by Walasek and Brown (2015) also hold on a cross-national level. Cross-sectional evidence for the negative socio-economic consequences of inequality is found at both national and international levels of analysis (see Wilkinson and Pickett 2009 for a review). Thus if income inequality is associated with more interest in positional goods, the relationship between internet searches and inequality level should hold using data on different nations. Importantly, this analysis is not possible using Google Correlate, which is currently limited to state-by-state comparisons in the US, and time-series analyses across different countries. Instead, we use Google Trends (https://www.google.co.uk/trends/) to compare search frequencies for specific terms in different nations. Google Trends reverses the way in which Google Correlate operates. Providing Google Trends with a list of internet queries produces a time series of their relative search frequency. If the findings of Walasek and Brown (2015) generalize to a national level, these frequencies should be correlated with income inequality, even when the effects of income are controlled for.

The second goal of the following paper is to extend the findings of Walasek and Brown (2015) and to address a potential limitation of their initial findings. As input for Google Correlate, the authors used residuals obtained from regressing income inequality (GINI coefficient) on various control variables, including log of mean income. However, controlling for mean income does not eliminate the possibility that internet searches for status goods differ as a function of the proportion of people with high incomes, which will be correlated with inequality. It is therefore important to exclude the possibility that apparent effects of inequality on concern with positional goods reflects non-linear effects of income on consumer spending.

## Study I

The objective of Study 1 is to extend the work of Walasek and Brown (2015) to the national level. We test whether search term frequencies for luxury brands are associated with the level of income inequality across different countries. If income dispersion promotes status competition, we should observe that people in more unequal countries search more often for luxury brands, even when income level is controlled for.

### Methods and Variables

We obtained GINI coefficients for the year 2009 from the International Database of Income Inequality (Solt 2009) in order to ensure that the inequality measures were as comparable as possible. Income data for the same year were acquired from the World Bank Data (2009). In order to control for earnings of the richest members of the population, we used country-level data on household consumption per capita by income groups (http://en.wikipedia.org/wiki/User:Pristino/List_of_countries_by_income_groups_of_household_consumption_per_capita), obtained from the World Development Indicators. These data represent household final consumption expenditure (HFCE) expressed in purchasing power parity (PPP) terms. This allows us to compare spending of the top 10 % of the countries’ populations in constant 2005 international dollars.

### Search Terms Selection and Google Trends

Google Trends calculates the relative search frequency of a pre-determined list of words and phrases. Up to five terms can be submitted to Google Trends simultaneously. In order to obtain the top five luxury brands, we conducted an online survey on Amazon Mechanical Turk, asking 275 respondents to list ten consumer brands. Here we only focus on a third of this sample,^1^ who were explicitly asked:

In the following task, we would like you to list ten brands. We are interested in *high status* brands/makes/labels of any consumer products that you can think of. High status refers to brands that are associated with high income and wealth.

Each participant in the online survey was rewarded with $0.50 for their time. We identified the top five brands that were most frequently mentioned by our participants (excluding automobile brands). Our final top five companies were “Gucci”, “Louis Vuitton”, “Rolex”, “Prada”, and “Chanel”.^2^

All five terms were entered simultaneously into Google Trends. Their relative frequency was calculated for the period between January 2009 and December 2014. Only average scores for each country were saved.

## Results and Discussion

We first regressed the relative frequency of the searches for the five luxury brands on log of mean income, income inequality (GINI coefficient), and their interaction. All variables were standardized prior to analysis. Data were available from 99 nations in total, and the results are summarized in Table 1. In line with the prediction of the social rank hypothesis, the relative frequencies with which people search for “Gucci”, “Louis Vuitton”, “Rolex”, “Prada” and “Chanel” in 99 countries increase as a function of income and income inequality, although the effect of the latter is only marginally significant.

**Table 1:** Cross-national regression results

Predictor	β	*t*(95)	*p*
Income inequality (GINI)	.18	1.82	.074
Log(mean income)	.64	5.94	<.001
Income inequality (GINI) * Log(mean income)	.18	2.20	.047

Adjusted R^2^ of the model was .28

**Fig. 1:**
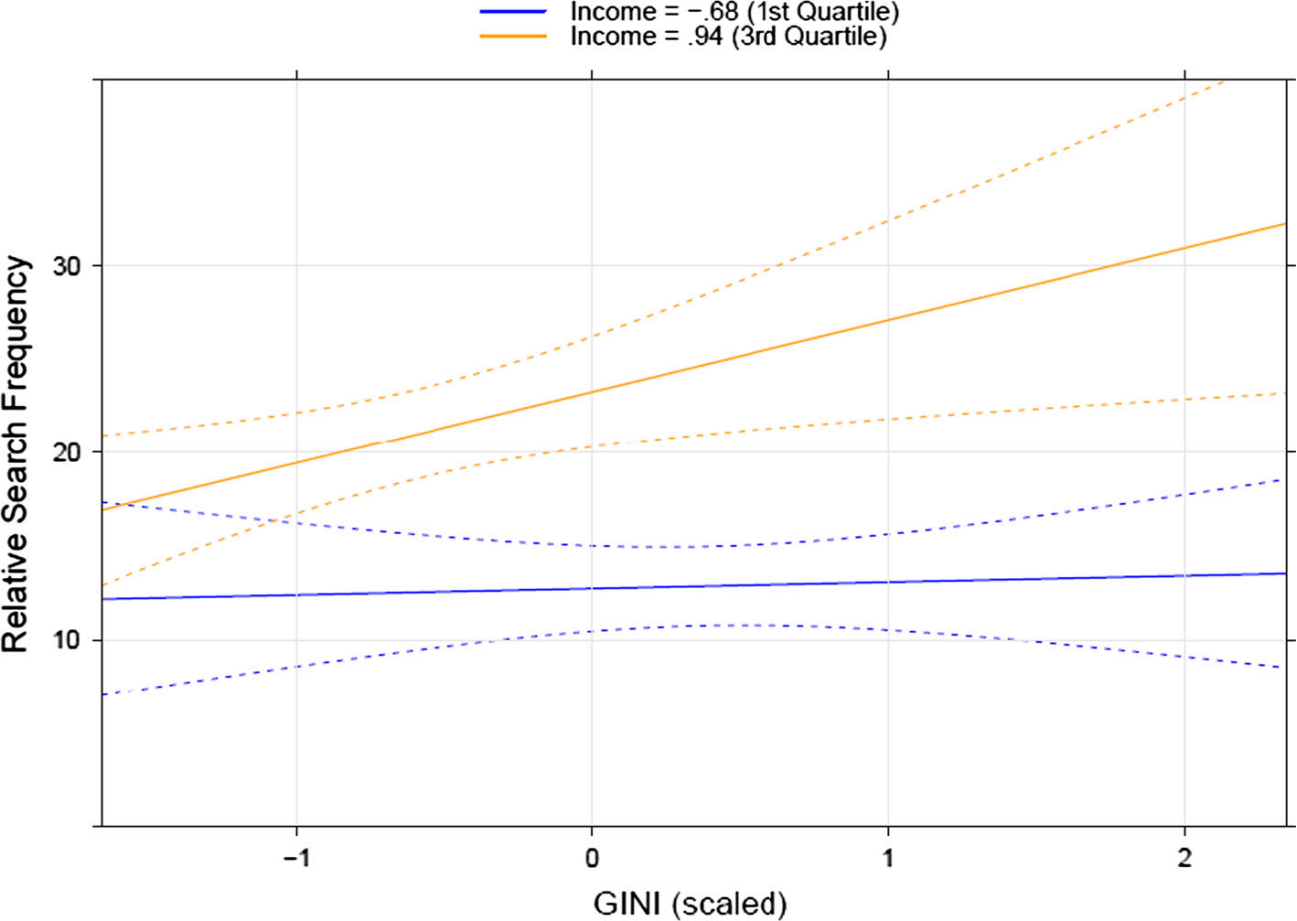
Relationship between GINI and the relative search term frequency for the top five luxury brands. *Lines* represent model predictions with income held constant

Importantly, we also found a significant interaction between nations’ income and inequality. This is consistent with the literature on the nation-level consequences of income inequality, which shows that the effect of income inequality is stronger in wealthier countries (e.g., Wilkinson and Pickett 2009). This relationship is shown in Fig. 1, where the two lines represent model’s predictions when income is held constant at low (1st quartile) and high (3rd quartile) value.

Together these results extend the findings reported by Walasek and Brown (2015), showing that interest in positional goods is associated with income inequality on the national level. However, it is possible that these results are still driven by the effect of income, if for example people’s spending on luxury goods varies non-linearly as a function of their earnings. If mean income is constant across two societies which differ in income inequality, there will inevitably be a higher proportion of very rich individuals (i.e., people with income over a certain threshold) in the unequal society. If spending on positional goods goes up non-linearly with income, such that richer people spend a greater proportion of their income on positional goods, an apparent effect of inequality could reflect the higher proportion of rich people in the unequal society. Related phenomena have been much explored in, for example, the literature concerned with income inequality and health (Deaton 2003; Gravelle et al. 2002; Kondo et al. 2009).

**Fig. 2:**
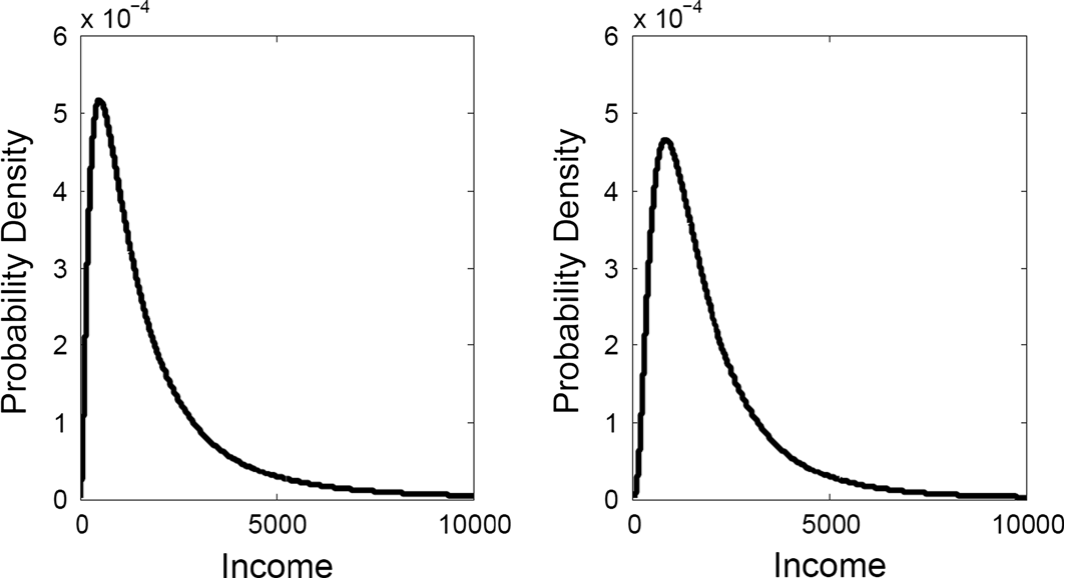
Two exemplar log-normal income distributions with mean income of $2000. GINI coefficients for these distributions are .5 (*left panel*) and .4 (*right panel*)

To illustrate the problem, consider two different societies, within income distributions as shown in Fig. 2.

The left-hand panel shows a log-normal income distribution with a mean of 2000 and a GINI coefficient of .50. The right-hand panel shows a more equal distribution, constructed to have the same mean (2000) but a GINI coefficient of .40. Suppose that the spending on consumer goods of an individual in each of the distributions, S_*i*_, increases as a power function of *i*’s income, w_i_^*a*^ (*a* > 1). It follows that the total spending on consumer goods summed over individuals will be larger in the less equal society. For example, if *a* is 1.5, spending will be 13 % higher in the more unequal society. If *a* is 1.1 or 2.0, the percentages are 2 and 40 % respectively.

The spending patterns of the richest few percent of a society seem unlikely to explain the patterns observed in Google searches of the whole population. There are more than twice as many individuals earning over 8000 in the left-panel distribution as in the right-panel distribution, and 56 % more individuals earning over 6000, but these individuals make up only a small percentage of the population. Nonetheless, we address the possibility that the results simply show that status-related searches are driven by the larger proportion of richer people in an unequal society. To exclude this possible confound in our analysis, we included spending of the top 10 % of the population in our regression model. Specifically, we regressed the relative search frequency for five luxury brands on income inequality, log of income (and their interaction), and spending of the richest 10 % (log transformed) of nations’ population. The results are presented in Table 2.

**Table 2:** Cross-national regression results

Predictor	β	*t*(74)	*p*
Income inequality (GINI)	.17	1.54	.128
Log(mean income)	.73	3.01	.004
Income (top 10 %)	−.05	−.25	.807
Income inequality (GINI) * Log(mean income)	.24	2.14	.036

All variables are centred

Adjusted R^2^ of the model was .29

**Fig. 3:**
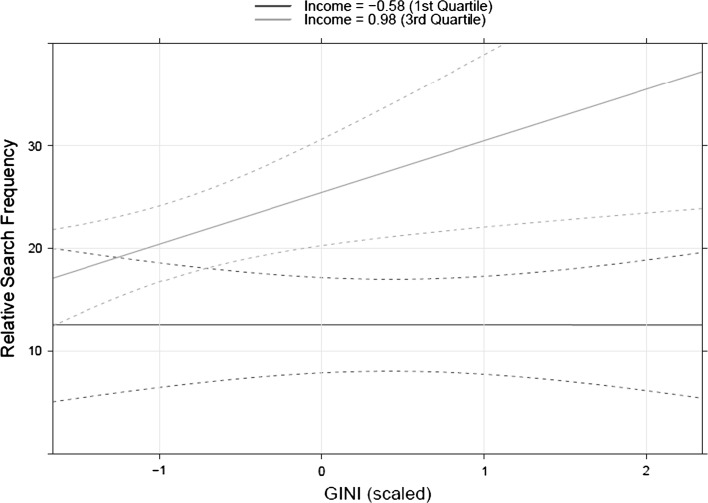
Relationship between GINI and the relative search term frequency for the top five luxury brands. *Lines* represent model predictions with income and earnings of the richest 10 % of the population held constant

The results show that the relative search frequency for luxury brands increases with income, but not with income inequality or spending of the richest 10 % of the population. Crucially, we again find a significant interaction of income and inequality, which suggests that internet searches for luxury brands were more common among the richer countries. This interaction, presented in Fig. 3, clearly shows that this is the case. Here we fix income at 1st and 3rd quartile, while holding the spending of the top 10 % constant at its median value.

In sum, this study extends the findings reported by Walasek and Brown (2015), showing that relative search frequency for high status international brands is higher in countries with higher levels of income inequality. Notably, this association is stronger in well-developed countries.

## Study II

In Study 1, we demonstrated that people’s interest in positional goods is higher in nations with higher level of income inequality. We have also argued that this effect is not driven by a non-linear relationship between earnings and interest in positional goods. However, it is still possible that the results reported by Walasek and Brown (2015) could be influenced by the larger number of wealthy people in unequal US states. In the following study, we therefore extend the results reported in Walasek and Brown and test their robustness by controlling for the income of the richest members of the population.

### Methods

Replicating the methodology of Walasek and Brown (2015), we regressed state-level income inequality (GINI coefficient) on mean income (log), total population, percent foreign born residents, and the percent of urban population. These data are 5-year estimates available from the U.S. Census Bureau (2010, 2012a, b, c, d). Additionally, we included the proportion of population earning more than $100,000 US dollars per year, obtained from the U.S. Census Bureau ( 2012b), as a predictor in the analysis. Equation 1, summarizes the complete model.

1 $$\begin{aligned} & Gini_{i} \sim \beta_{0} + \beta_{1} \ln \;\left( {income_{i} } \right) + \beta_{2} state\,population_{i} + \beta_{3} urban\,population_{i} \\ &\quad + \beta_{4}\;foreign\,born_{i} + \beta_{5}\;proportion\,earning\,above\,100k_{i} \\ \end{aligned}$$

Standardized residuals of the model were saved and submitted to Google Correlate on the 10th of April, 2015. We used both positive and negative residuals to generate lists of search term for which the relative frequency of occurrence correlates the most with our measure of residual income inequality. Google Correlate produces up to 100 search terms with a Pearson’s *r* of at least .6, and we saved the top 40.

## Results and Discussion

Table 3 shows the results of the regression model from Eq. 1. Table 4 lists the top forty search terms that correlate positively and negatively with residual income inequality.

**Table 3:** Regression results for Eq. 1

Predictor	β	*t*(44)	*p*
Log(mean income)	−1.54	−1.80	.079
Percent foreign born residents	.36	1.22	.230
State population	.45	2.73	.009
Percentage of the population in urban areas	.05	.23	.818
Percentage of the population earning more than $100,000 a year	1.19	1.45	.155

Adjusted R^2^ of the model was .52

**Table 4:** Top 40 search terms that correlate the most (positively and negatively) with residual income inequality

Current study				Walasek and Brown (2015) results			
*r*	Positive *r*	*r*	Negative *r*	*r*	Positive *r*	*r*	Negative *r*
.76	Paula zahn	−.71	Smart cast	.78	Ralph Lauren mens	−.72	Mekenna
.75	Dix bay	−.71	Ram?	.77	Ralph	−.72	Flower names
.75	Little dix bay	−.71	Radeon 7950	.76	Ralph Lauren womens	−.71	Blizzard entertainment
.75	Fur vests	−.70	Chicken bake	.76	Paula Zahn	−.71	Stumbler
.75	Vineyardvines.com	−.70	Word dictionary	.75	Fur vests	−.71	Chicken bake
.74	Bunny williams	−.70	Heroes of	.75	David Yurman earrings	−.71	Mt Pinatubo
.74	Jumby bay antigua	−.70	Action camera	.75	Vineyardvines.com	−.71	Pirate talk
.74	Little dix	−.70	Flower names	.75	Brown suede	−.71	Top view
.74	Bacon egg and cheese	−.69	Diablo 3 monk	.75	Ralph Lauren blue	−.70	Chick flick movies
.73	Ralph	−.69	Mekenna	.75	Fig trees for sale	−.70	Heroes of
.73	Ralph lauren mens	−.69	Tactic	.75	Dix Bay	−.70	Diablo
.73	Martha moxley	−.69	Diablo	.75	Little Dix Bay	−.70	Firefox add
.73	St thomas ritz	−.68	Blizzard entertainment	.75	Yurman rings	−.70	Barfing
.73	Woman attacked	−.68	Firefox add	.74	Designer rain boots	−.70	Super moist
.73	Well appointed house	−.68	Trundle build	.74	Maxima spoiler	−.70	Tactic
.73	Well appointed	−.68	Skarner build	.74	Jumby Bay Antigua	−.69	Ram?
.73	Charlotte moss	−.68	Super funny	.74	Ralph Lauren	−.69	Spamcop
.72	Colefax and fowler	−.68	Postage price	.74	David Yurman rings	−.69	Lemon bars recipe
.72	Maxima spoiler	−.68	Death adder	.74	Ralph Lauren baby	−.69	Word dictionary
.72	Woman attacked by chimp	−.68	Server location	.74	Navy blazer	−.69	Battery care
.72	Palms turks and caicos	−.68	Battery care	.74	Woman attacked	−.69	Extractors
.72	Ralph lauren womens	−.68	Internet ip	.73	St Thomas Ritz	−.69	Radeon 7950
.72	David yurman earrings	−.68	Zilean build	.73	Fibroadenoma	−.69	Pinatubo
.72	Ralph lauren blue	−.67	Cassiopeia	.73	Penny loafer	−.69	Postage price
.72	Brown suede	−.67	Mousehunt	.73	David Yurman	−.69	Komodo
.72	Hibachi restaurants	−.67	Amd a10	.73	Yurman	−.69	5 gen
.72	Dominick dunne	−.67	Battlenet	.73	Ralph Lauren boys	−.69	Internet IP
.71	Matouk bedding	−.67	Mt pinatubo	.73	Johnston and Murphy	−.69	Transfer windows
.71	Ritz carlton st thomas	−.67	Lemon bars recipe	.73	Little Dix	−.68	Smart cast
.71	Attacked by chimp	−.67	Brushless	.73	Yurman earrings	−.68	Origami ninja
.71	Palms turks	−.67	Dota 2 release date	.73	Well appointed house	−.68	Moist chicken
.71	Curtain bluff	−.67	Dota 2 release	.73	Yurman.com	−.68	No post
.71	Bass loafers	−.67	Legend of the guardians	.73	Bass loafers	−.68	Pony beads
.71	Eddie ross	−.67	Oh my goddess	.73	Driving loafers	−.68	Name definitions
.71	Jalousie plantation	−.67	Graphics processor	.73	Worth collection	−.68	Crystal disk
.71	Le toiny	−.67	Version pokemon	.73	Champagne punch	−.68	Viking sewing
.71	Coren moore	−.67	How to use a semicolon	.73	Seersucker blazer	−.68	Sanitizing
.71	Serena and	−.67	Light diffuser	.73	Fatal attraction	−.68	Viking sewing machine
.71	Juliska	−.67	Night fury	.73	Tibi dresses	−.68	Action camera
.71	Fibroadenoma	−.67	Barfing	.73	David Yurman jewelry on sale	−.68	Obituary California

This table shows the results of the current study along with the results reported by Walasek and Brown (2015)

From inspecting search terms in Table 4, it is immediately evident that there are many status-related goods and brands among the search terms that positively correlate with residual income inequality. Indeed, the results are very similar to those reported by Walasek and Brown (2015)—brands and goods such as Ralph Lauren, Dix Bay, Brown suede, Bass loafers, well-appointed house, and fur vests occur in both lists. A considerable overlap can be also seen among the negatively correlated terms. Here searches for chicken bake, tactic, battery care, and lemon bar recipes co-occur. Consistent with Walasek and Brown, negatively correlated terms do not seem to include any luxury brands or lavish consumer products.

For robustness, we conducted the same analysis using residuals obtained from a model where the proportion of people earning more than $100,000 was replaced with the proportion of people earning more than $50,000 and $200,000. Interestingly, submitting the resulting residuals into Google Correlate does not produce any interpretable output—the algorithm does not find more than two search terms with correlation above .6.

As a further test of robustness, we reversed our analysis and used residual income (after controlling for inequality) as input for Google Correlate. We regressed state-level log of income on income inequality and the three control variables: state population, percent of urban population and percent of foreign population. Residuals from this analysis were submitted to Google Correlate, and the resulting search terms are listed in Table 5. Also, in Table 5 we summarize the search terms that were generated when we performed the same regression on the proportion of people earning over $100,000. Table 6 shows the results of the two regression analyses.

**Table 5:** Search terms correlated with residual income and proportion of the population earning more than $100,000

Residual income				Residual of the proportion earning more than $100,000			
*r*	Positive *r*	*r*	Negative *r*	*r*	Positive *r*	*r*	Negative *r*
.73	Goalie camp	−.80	Megashare.com	.72	Dell studio 15	−.75	Megashare.com
.72	Recessed lights	−.78	Megashare.info	.72	Bare necessities coupon	−.74	Megashare.info
.72	Bare necessities coupon	−.78	Free disney movies	.72	Recessed light	−.74	Free disney movies
.72	Recessed light	−.77	Mp280	.70	Recessed lights	−.74	Mp280
.72	Dell studio 15	−.76	The walking dead season 4 episode 1	.70	Erm	−.74	The walking dead season 4 episode 1
.71	Stair runners	−.75	Walking dead season 4 episode 1	.70	Aprilaire 400	−.73	Walking dead season 4 episode 1
.71	Health forms	−.75	http://192.168.o.1	.69	Mefloquine	−.73	http://192.168.o.1
.70	V-neck sweater	−.74	How do i check	.68	Health forms	−.73	How do i check
.70	Recessed	−.74	Computer repair	.68	German school	−.72	Computer repair
.70	Erm	−.74	Up images	.68	Ovechkin	−.71	Up images
.70	Zip sweater	−.73	Cheap business cards	.68	Brendan sullivan	−.71	Cheap business cards
.70	Toys to grow on	−.73	http://192.168.o.1	.68	Ovechkin goal	−.71	http://192.168.o.1
.70	Aprilaire 400	−.73	Public restroom	.68	Male female ratio	−.71	Public restroom
.70	Safety gate	−.73	How do i qualify for	.68	Larry levine	−.70	How do i qualify for
.70	Toys to grow	−.73	Chiuaua	.68	Toys to grow on	−.70	Chiuaua
.69	Driveway sealing	−.73	Chihuahua mix	.68	Recessed	−.70	Chihuahua mix
.69	Iceland tourism	−.73	How do i qualify	.67	V-neck sweater	−.70	How do i qualify
.69	One step ahead	−.73	Watch cars	.67	Recessed lighting	−.70	Watch cars
.69	Saving for college	−.72	Beverly hills chihuahua 2	.67	Cadette	−.69	Beverly hills chihuahua 2
.69	Car coat	−.72	Fatsickandnearlydead	.67	Toys to grow	−.69	Fatsickandnearlydead
.69	Stair runner	−.72	Isuzu amigo	.67	Goalie camp	−.69	Isuzu amigo
.69	Politburo	−.72	The walking dead season 4 episode	.67	One step ahead	−.69	The walking dead season 4 episode
.69	Us Russia	−.72	2 player	.67	Zip sweater	−.69	2 player
.69	Recessed lighting	−.72	Freemake video	.67	Triclimate	−.69	Freemake video
.69	Company store coupons	−.72	What is the best internet	.66	How much mortgage can i afford	−.69	What is the best internet
.68	Carpet runner	−.71	Printer for sale	.66	Lighting direct	−.69	Printer for sale
.68	Erg	−.71	Drawings tumblr	.66	Ira income limits	−.69	Drawings tumblr
.68	Lighting direct	−.71	How to draw a baby	.66	Erg	−.68	How to draw a baby
.68	Ovechkin	−.71	Canon mp280	.66	Iceland tourism	−.68	Canon mp280
.68	Arts and letters daily	−.71	Draw a baby	.66	Saving for college	−.68	Draw a baby
.68	Hockey showcase	−.71	Resume creator	.66	Us Russia	−.68	Resume creator
.68	Cheese of the month	−.71	www.netflix.com/activate	.66	Triclimate jacket	−.68	www.netflix.com/activate
.68	Mortgage can i afford	−.71	Ink refills	.66	Alex ovechkin	−.68	Ink refills
.68	Ira income limits	−.71	Marvel games	.66	Exchange 5.5	−.68	Marvel games
.68	Snow melter	−.71	Freemake	.66	Allocations	−.68	Freemake
.68	Turtleneck sweater	−.71	Pirate bay.com	.66	Bolger	−.67	Pirate bay.com
.68	Triclimate	−.71	Text faces	.66	Bathroom design	−.67	Text faces
.68	How much mortgage can i afford	−.71	Qualify	.66	Roth ira income limits	−.67	Qualify
.68	Cadette	−.71	Qualify for	.66	Lands end promotion	−.67	Qualify for
.67	Lands end promotion	−.71	Girl images	.66	Johns hopkins cty	−.67	Girl images

**Table 6:** Results of two regression analyses when log(income) (top panel) and proportion of people earning more than $100,000 (bottom panel) are used as the dependent variables

(a) Dependent variable: log(income)			
Predictor	β	*t*(45)	*p*
Percent foreign born residents	.72	3.61	.001
State population	−.18	−1.23	.224
Percentage of the population in urban areas	.18	1.06	.294
Income inequality (GINI)	−.22	−1.86	.070

(b) Dependent variable: proportion earning over 100 K			
Predictor	β	*t*(44)	*p*
Percentage of foreign-born population	.73	3.48	.001
State population	−.23	−1.53	.134
Percentage of the population in urban areas	.14	.78	.442
Income inequality (GINI)	−1.89	−1.51	.137

^a^Adj. R^2^ = .52

^b^Adj. R^2^ = .46

From inspecting the search terms, it is clear that when we control for the level of income inequality (among other variables), state-level income is not associated with searches for positional goods. Terms listed in Table 5 are at stark contrast with those in Table 4—it is clear that these internet searches are not related to social status. The list includes searches for recessed lighting, driveway sealing, or bare necessities coupon. This represents a strong test of the hypothesis that inequality, rather than income, is related to searches for positional goods.

## General Discussion

In two studies we found that internet search terms related to positional goods are relatively more frequent in regions with higher levels of income inequality. This finding is consistent with the social rank hypothesis, which maintains that when income becomes a better signal of one’s position within a social hierarchy, people become more concerned with goods and brands that signal social status. In Study 1, we showed that the relative search frequency for five well-known luxury brands is higher in nations with higher income inequality. This association is stronger among well-developed countries, here indexed by higher income. We also demonstrated that this relationship could not be explained by the spending tendencies of the wealthiest members of a society. In Study 2, we showed that the same tendency previously reported within a nation is unlikely to be driven by the consumption of the richest members of the society. Inequality remains positively associated with status-seeking even when we control for both mean income and spending among the wealthiest individuals. At the same time, we demonstrated that income alone is not associated with internet searches for luxury goods and brands. Together, results both address some potential limitations of the previous work (Walasek and Brown 2015) and present new support for the notion that income inequality leads to higher concern with social status.

We undertook several steps to avoid potential pitfalls inherently associated with correlational research. In order to avoid the risk of spurious correlations, we used regression residuals as input for Google Correlate, and were therefore able to control for a range of confounding variables. Although it is plausible that wealthier individuals spend relatively more of their disposable income on status-related goods, we were able to show that this tendency is unlikely to explain internet searches in unequal regions. Consistent with the broad literature on income inequality, we believe that searches for positional goods are likely to be higher for all levels of wealth and income. These results are consistent with those of other authors, who found that even among the wealthiest individuals, status anxiety is higher in societies with higher overall level of income inequality (Layte and Whelan 2014). Our replication on the cross-national level further shows that it is inequality, rather than income, that determines status-seeking behaviours. In line with previous findings, we showed that searches for luxury brands such as Prada or Hermes are more common in countries where both average income and income inequality are high.

The findings at cross-country level may seem surprising, given that our five brands were identified by survey respondents located in the U.S. These brands were nonetheless known among the internet users in different nations. Indeed, if the search frequency for these labels was too low, Google Trends would be unable to produce reliable time-series data. Similarly, if the access to internet was limited in one country, we would not be able to obtain enough data from Google Trends. It is still possible that only the richest individuals who live in urban areas have access to internet in some of the poorer countries. Although this is likely to be the case, we excluded the possibility as far as possible by controlling for income and its distribution.

In interpreting our data, we do not exclude the possibility of bi-directional causation. It is plausible that a large personal investment in status-seeking can lead to a worsening divide between the poor and the rich. As previous research suggests, inequality is associated with over-spending and higher likelihood of becoming indebted (Alvarez-Cuadrado and Attar 2012; Cynamon and Fazzari 2013; Heffetz 2011). Individuals who prefer to spend their income on status-competition through the consumption of positional goods, are unlikely to truly improve their personal circumstances.

To conclude, our findings show that in regions with high income inequality, people are more status-seeking. Specifically, they are more likely to spend time searching for positional goods and luxury brands on the internet. These results complement previous work showing that status-consumption is rife in developed and highly unequal regions. Further research needs to focus on the individual and societal consequences of the pre-occupation with status-seeking.

## Footnotes

1 For purposes unrelated to this study, we asked the remaining participants to list either any brands or brands that are related to low social status.

2 For robustness, we repeated the analysis with the subsequent five popular brands (Coach, Armani, Versace, Burberry, Hermes). All analyses yielded the same results.

## References

[CR1] Alvarez-Cuadrado, F., & Attar, M. E. (2012). Income inequality and saving. In *IZA discussion paper. no 7083*.

[CR2] Bowles S, Park Y (2005). Emulation, inequality, and work hours: Was Thorsten Veblen right?. Economic Journal.

[CR3] Bricker, J., Ramcharan, R., Krimmel, J., Bricker, J., Ramcharan, R., & Krimmel, J. (2014). *Signaling status: The impact of relative income on household consumption and financial decisions (FEDS working paper)*.

[CR4] Brown, G. D. A., Boyce, C. J., & Wood, A. M. (2014). Inequality, well-being, and social rank: An income increase buys more life satisfaction in more equal countries. *Manuscript Submitted for Publication*.

[CR5] Chao A, Schor JB (1996). Empirical tests of status consumption: Evidence from women’s consmetics. Journal of Economic Psychology.

[CR6] Cynamon, B. Z., & Fazzari, S. M. (2015). Inequality, the Great Recession, and Slow Recovery. *Cambridge Journal of Economics*. doi:10.1093/cje/bev016.

[CR7] Daly M, Boyce C, Wood A (2015). A social rank explanation of how money influences health. Health Psychology.

[CR8] Deaton AS (2003). Health, inequality, and economic development. Journal of Economic Literature.

[CR9] Drechsel-Grau M, Schmid KD (2014). Consumption-savings decisions under upward looking comparisons: Evidence from Germany, 2002–2011. Journal of Economic Behavior & Organization.

[CR10] Gravelle H, Wildman J, Sutton M (2002). Income, income inequality and health: What can we learn from aggregate data?. Social Science and Medicine.

[CR11] Heffetz O (2011). A test of conspicuous consumption: Visibility and income elasticities. Review of Economics and Statistics.

[CR12] Hirsch F (1977). Social limits to growth.

[CR13] Kondo N, Sembajwe G, Kawachi I, van Dam RM, Subramanian SV, Yamagata Z (2009). Income inequality, mortality, and self rated health: meta-analysis of multilevel studies. BMJ: British Medical Journal.

[CR14] Layte R, Whelan CT (2014). Who feels Inferior? A test of the status anxiety hypothesis of social inequalities in health. European Sociological Review.

[CR15] Perugini, C., Jens, H., & Collie, S. (2015). Inequality, credit expansion and financial crises. *Cambridge Journal of Economics*. doi:10.1093/cje/beu075.

[CR16] Solt F (2009). Standardizing the world income inequality database. Social Science Quarterly.

[CR17] The World Bank. (2009). Net income from abroad (current US$). Retrieved from http://data.worldbank.org/indicator/NY.GSR.NFCY.CD?page=1.

[CR18] U.S. Census Bureau. (2010). Urban and rural: Area facts. 2010 census congressional district summary file (113th Congress). Retrieved from http://factfinder.census.gov/.

[CR19] U.S. Census Bureau. (2012a). GINI index of income inequality: 2008–2012 American Community Survey 5-year estimates. Retrieved from http://factfinder.census.gov/bkmk/.

[CR20] U.S. Census Bureau. (2012b). Household income in the past 12 months (in 2012 inflation-adjusted dollars): 2008–2012 American Community Survey 5-year estimates. Retrieved from http://factfinder.census.gov/bkmk/table/1.0/en/ACS/.

[CR21] U.S. Census Bureau. (2012c). Selected social characteristics in the United States: 2008–2012 American Community Survey 5-year estimates. Retrieved from http://factfinder.census.gov/bkmk/table/1.0/en/ACS/.

[CR22] U.S. Census Bureau. (2012d). Total population: 2008–2012 American Community Survey 5-year estimates. Retrieved from http://factfinder.census.gov/bkmk/table/1.0/en/ACS/.

[CR23] Walasek, L., & Brown, G. D. A. (2015). Income inequality and status seeking: searching for positional goods in unequal U.S. states. *Psychological Science*, *29*, 1–7. 10.1177/0956797614567511 25792131

[CR24] Wilkinson RG, Pickett KE (2009). The spirit level: Why equality is better for everyone.

